# Genome-Wide Analysis of C2H2 Zinc-Finger Family Transcription Factors and Their Responses to Abiotic Stresses in Poplar (*Populus trichocarpa*)

**DOI:** 10.1371/journal.pone.0134753

**Published:** 2015-08-03

**Authors:** Quangang Liu, Zhanchao Wang, Xuemei Xu, Haizhen Zhang, Chenghao Li

**Affiliations:** 1 State Key Laboratory of Tree Genetics and Breeding, Northeast Forestry University, Harbin, Heilongjiang, People’s Republic of China; 2 Library of Northeast Forestry University, Harbin, Heilongjiang, People’s Republic of China; National Institute of Plant Genome Research, INDIA

## Abstract

**Background:**

C2H2 zinc-finger (C2H2-ZF) proteins are a large gene family in plants that participate in various aspects of normal plant growth and development, as well as in biotic and abiotic stress responses. To date, no overall analysis incorporating evolutionary history and expression profiling of the C2H2-ZF gene family in model tree species poplar (*Populus trichocarpa*) has been reported.

**Principal Findings:**

Here, we identified 109 full-length C2H2-ZF genes in *P*. *trichocarpa*, and classified them into four groups, based on phylogenetic analysis. The 109 C2H2-ZF genes were distributed unequally on 19 *P*. *trichocarpa* linkage groups (LGs), with 39 segmental duplication events, indicating that segmental duplication has been important in the expansion of the C2H2-ZF gene family. Promoter *cis*-element analysis indicated that most of the C2H2-ZF genes contain phytohormone or abiotic stress-related *cis*-elements. The expression patterns of C2H2-ZF genes, based on heatmap analysis, suggested that C2H2-ZF genes are involved in tissue and organ development, especially root and floral development. Expression analysis based on quantitative real-time reverse transcription polymerase chain reaction indicated that C2H2-ZF genes are significantly involved in drought, heat and salt response, possibly via different mechanisms.

**Conclusions:**

This study provides a thorough overview of the *P*. *trichocarpa* C2H2-ZF gene family and presents a new perspective on the evolution of this gene family. In particular, some C2H2-ZF genes may be involved in environmental stress tolerance regulation. *PtrZFP2*, *19* and *95* showed high expression levels in leaves and/or roots under environmental stresses. Additionally, this study provided a solid foundation for studying the biological roles of C2H2-ZF genes in *Populus* growth and development. These results form the basis for further investigation of the roles of these candidate genes and for future genetic engineering and gene functional studies in *Populus*.

## Introduction

Zinc-finger proteins (ZFPs) are the largest family of transcription regulators in plants [1]. The term “zinc finger” (ZF) refers to a protein domain, in which a zinc ion is surrounded by cysteine (Cys) residues and/or the histidine (His) residues to stabilize its three-dimensional structure, which comprises a two-stranded antiparallel beta sheet and a helix. Based on the number and location of the Cys and His residues, the ZFPs are divided into different types: C2H2, C2HC, C2HC5, C2C2, CCCH, C3HC4, C4, C4HC3, C6, and C8 [2,3]. The first plant-specific ZFP was identified in *Petunia* and interacts with the promoter region of the 5-enolpyruvylshikimate-3-phosphate synthase gene (EPSPS) [4]. Subsequently, further zinc-finger transcription factors (TFs) have been identified in other plants and their contributions to important biological processes during vegetative growth, reproductive development and stress responses have been noted [5–8].

The C2H2 ZFPs, also called the TFIIIA-type ZFPs, can be represented as X2-Cys-X(2–4)-Cys-X12-His-X(3–5)-His, where X represents any amino acid and where the precise spacing varies between the two cysteines and between the two histidines [9]. Among the ZFP types, C2H2-ZFPs are one of the most widespread transcription factor families in eukaryotes [10]. *In silico* analysis has shown that C2H2-ZF genes represent ~3% of all genes in mammals, ~2.3% in Diptera and ~0.8% in *Saccharomyces cerevisiae* [11,12]. In plants, the C2H2 zinc-finger gene family is large. There are 176, 189 and 124 members in *Arabidopsis*, rice and foxtail millet, respectively [13–15]. Compared with other eukaryote C2H2-ZFPs, there are two main structural features in most of the plant C2H2 ZFPs. First, in plant multiple-fingered C2H2-ZFPs, the zinc-finger domains are separated by long spacers that vary in length and sequence, whereas the C2H2-ZFPs of yeast and animals are mostly clustered and separated by a short spacer (six to eight amino acids) [16,17]. Second, most plant C2H2-ZFPs include an invariant sequence, QALGGH, located inside the zinc-finger helices, whereas yeast and animal C2H2-ZFPs do not have this motif [1].

C2H2-ZFPs differ widely in their structures and functions. Many ZFPs function as TFs, and although these proteins are thought to mainly interact with DNA or chromatin, some are also thought to bind to RNA and proteins [18–20]. Therefore C2H2-ZFPs not only participate in transcriptional regulation, but also act in RNA metabolism and in other biological activities. They are involved in a wide range of processes, including plant growth and development, and in biotic and abiotic stress responses in plants. A number of C2H2-ZFPs are involved in trichome initiation, seed germination, floral organogenesis, primary microRNA biogenesis and heat stress in *Arabidopsis* [21–25]; cold, drought, oxidative and salt stress in rice [26,27]; cold and drought stress in soybean [28]; pathogen defense in *Capsicum annuum* [29]; and osmotic, cold and mechanical stress in poplar [30]. These studies showed that C2H2-ZFPs are likely to be associated with multiple physiological processes and stress responses.

Poplar (*Populus trichocarpa*) is a valuable forest resource, making it an important commercial and ecological species [31]. *P*. *trichocarpa* is also a model plant, whose whole genome sequence is available [32]. Compared with the extensive studies of C2H2-ZF genes in many other plant species, little research has been conducted in *Populus* so far. Therefore, the genome-wide identification and expression analysis of the C2H2-ZF gene family is important. Here, we report a systematic study of this gene family. We identified 109 C2H2-ZF genes and analyzed their phylogenetic relationships, chromosomal locations, gene structures, conserved protein motifs and promoter *cis*-elements. The expression profiles of this gene family in different tissues and organs of *Populus*, and under biotic and abiotic stress conditions, were analyzed using data from heatmap and quantitative real-time reverse transcription polymerase chain reaction (qRT-PCR) analyses. Our results provided a subset of potential candidate genes that may be used for future genetic engineering and gene functional studies in *Populus*.

## Materials and Methods

### Ethics Statement

No specific permissions were required for the described field studies. Our university, the Northeast Forestry University in Harbin, is not privately-owned. The field studies did not involve endangered or protected species.

### Identification and characteristics of the C2H2-ZF gene family

The genomic library, cDNA library and protein database of *P*. *trichocarpa* were downloaded from the Phytozome v10.0 (http://phytozome.jgi.doe.gov/pz/portal.html) and NCBI (http://www.ncbi.nlm.nih.gov/) databases. The hidden Markov model (HMM) profile of the C2H2-ZF gene family (protein family ID: PF00096) was downloaded from the Protein family database (Pfam 27.0, http://pfam.xfam.org/) [33]. All of the located sequences were further analyzed manually to confirm the presence of a C2H2-ZF domain (SM000355) using the SMART (http://smart.embl-heidelberg.de/) database [34]. Finally, the obtained genes were compared with the C2H2-ZF gene family in PlnTFDB v3.0 (http://plntfdb.bio.uni-potsdam.de/v3.0/) [35]. Closely related genes from *Arabidopsis* were obtained from the *Arabidopsis* Information Resource (TAIR, http://www.arabidopsis.org/index.jsp). WoLF PSORT (http://wolfpsort.org/) [36] was used to predict the subcellular localization of C2H2-ZFPs. The ExPasy site (http://web.expasy.org/protparam/) [37] was used to calculate the molecular weight and isoelectric point (pI) of the deduced polypeptides.

### Phylogenetic analysis

Multiple sequence alignment of the full-length protein sequences was performed using Clustal X (version 1.83) [38] and adjusted manually using BioEdit 7.1 software [39]. Phylogenetic analyses using the neighbor-joining method in MEGA 5.0 [40] and a bootstrap test carried out with 1000 iterations were performed to construct unrooted phylogenetic trees.

### Exon/intron structure analysis and identification of conserved motifs

The Gene structure display server (GSDS 2.0, http://gsds.cbi.pku.edu.cn/index.php) [41] was used to generate the exon/intron organization. The Multiple Expectation Maximization for Motif Elucidation (MEME) system (Version 4.9.1, http://meme.nbcr.net/meme/) was used to identify conserved motifs for each C2H2-ZF gene [42]. Structural motif annotation was performed using the Pfam and SMART tools.

### Chromosomal location

Genes were mapped on chromosomes by identifying their chromosomal locations provided in the PopGenIE v3 database (http://www.popgenie.org/) [43]. Physical map was constructed using Adobe Illustrator CS5 (Adobe Systems Incorporated). Blocks of the same color represent the homologous chromosome fragments. Genes were considered to have undergone segmental duplication if they were located on duplicated chromosomal segments [44]. Meanwhile, to search for potential duplicated *Populus* C2H2-ZF genes, MCScanX software (http://chibba.pgml.uga.edu/mcscan2/) was used [45], apart from them falling into duplicated blocks. Genes separated by five or fewer gene loci within a physical distance of 100 kb were considered tandem duplicates. A schematic view of the reorganization of homologous chromosomes segments was based on the most recent account of whole-genome duplication in *P*. *trichocarpa* [32]. Synonymous (Ks) and nonsynonymous substitution (Ka) rates were calculated according to previous study [46].

### Promoter *cis*-element analysis

Promoter sequences (2 kb upstream of the translation start site) of all C2H2-ZF genes were obtained from the Phytozome v10.0 database. PlantCARE (http://bioinformatics.psb.ugent.be/webtools/plantcare/html/) [47] was used to analyze the sequence of the C2H2-ZF gene promoters, and to predict and locate their *cis*-elements.

### Gene Ontology (GO) Annotation

The functional grouping of C2H2-ZFP sequences and the analysis of annotation data were executed using Blast2GO v3.0 [48]. Blast2GO annotation associates genes or transcripts with GO terms using hierarchical vocabularies. Genes are described in terms related to three categories of GO classification: biological processes, molecular functions and cellular components.

### exNorthern and exHeatMap analysis

The expression profile of each gene was obtained by evaluating its expressed sequence tag (EST) representation among 17 cDNA libraries derived from different tissues and/or developmental stages using the exNorthern tool of the PopGenIE v2 database (http://www.popgenie.org/). The 17 libraries were derived from several taxa of *Populus* [49]. The exHeatMap tool at PopGenIE v2 was used to visualize the heatmap of C2H2-ZF genes under different stress conditions. The heatmap data can be directly downloaded using the accession numbers of genes via the exHeatmap tool in the PopGenIE v2 database.

### Plant materials and stress treatments

Clonally propagated *P*. *trichocarpa* (genotype Nisqually-1) were cultured in half-strength Murashige and Skoog medium under long-day conditions (16 h light/8 h dark) at 23–25°C. Stress treatment conditions and sampling timings were conducted following the previous method with minor modification [50,51]. These clones were exposed to 150 mM mannitol, 200 mM sodium chloride (NaCl) and 42°C for drought, salinity and heat stress treatments, respectively. Young leaves and roots from three different plants were collected at different time points (0, 3, 6, 12 and 24 h) after treatment. Three biological replicates were performed for each stress treatment. Each experiment was repeated at least three times with independent sample preparation to obtain reproducible results. All samples were immediately frozen in liquid nitrogen and stored at −80°C until analysis.

### RNA isolation and qRT-PCR verification

The cetyltrimethylammonium bromide method, with minor modifications [52], was used to extract total RNA from roots and leaves. The first-strand cDNA synthesis was carried out with the ReverTra Ace qPCR RT Master Mix with a gDNA Remover Kit (TOYOBO, Osaka, Japan), in accordance with the manufacturer’s instructions. Primer Premier 5 was used to design primers with melting temperatures of 55–60°C, primer lengths of 18–25 bp, and amplicon lengths of 101–221 bp. Details of the primers are shown in S1 Table. SYBR Premix Ex Taq II (TaKaRa, Dalian, China) was used to perform qRT-PCR in accordance with the manufacturer’s instructions. Reactions were prepared in a total volume of 20 μL, containing 10 μL of 2×SYBR Premix, 2 μL of cDNA template, 6 μL of ddH_2_O, and 1 μL of each primer to make a final concentration of 10 μM. The *P*. *trichocarpa* actin gene (GenBank ID: XM_002298674) [53] was used as a reference gene. The PCR conditions and relative gene expression calculations were as previously described [54].

## Results

### Identification of C2H2-ZF genes in *Populus*

The HMM profile of the Pfam C2H2-ZF domain (protein family ID: PF00096) was used as the query to identify C2H2-ZF genes in the *Populus trichocarpa* genome. One hundred and twenty-one candidate C2H2-ZF genes were identified. All the C2H2-ZF candidates were analyzed manually using the SMART (SM000355) database to verify the presence of the C2H2-ZF domain. Finally, 109 C2H2-ZF genes were identified. Our result was roughly in agreement with PlnTFDB, where 110 members of the C2H2-ZF gene family were deposited for *Populus*. This number is less than the number present in the *Arabidopsis*, rice and foxtail millet genomes (176, 189 and 124, respectively) [13–15]. The C2H2-ZFPs have previously been named as AT-ZFP in *Arabidopsis thaliana*, hence we have named *Populus* proteins as PtrZFP (C2H2-ZFPs of *Populus trichocarpa*) [13].

The identified *P*. *trichocarpa* C2H2-ZF genes had molecular masses ranging from 17626.35 to 191195.46 Da. The encoded proteins varied from 161 to 1685 amino acids (aa), with an average of 404 aa. The pI values of the predicted proteins were varied; for example, PtrZFP59 had a pI of 4.75, whereas that of PtrZFP47 was 9.91. WoLF PSORT was used to predict the location of the predicted proteins in the plant cell. One hundred and three C2H2-ZFPs were predicted as nuclear proteins, four as chloroplast proteins and two as cytoplasmic proteins. SMART database was used to comfirm the number of C2H2 motifs. Based on the number of C2H2 motifs, the C2H2-ZF genes could be classified into two groups: a motif-rich group (PtrZFP16, 18, 67 and 70, each with 4–6 motifs) and a motif-poor group (for all the other proteins, the number of motifs varied from 1 to 3). The details of other characteristics of the nucleic acid and protein sequences are provided in S2 Table.

Of the identified 109 C2H2-ZF genes, 28 genes showed more than one gene model, which could be attributed to alternative splicing, in the Phytozome v10.0 database. These were *PtrZFP10*, *11*, *12*, *13*, *14*, *16*, *18*, *26*, *27*, *31*, *36*, *42*, *54*, *57*, *58*, *59*, *61*, *72*, *73*, *76*, *82*, *86*, *95*, *96*, *103*, *105*, *107* and *109*. *PtrZFP31*, *76* and *82* have the largest number (six) of transcripts. *PtrZFP16*, *26*, *27*, *54*, *86* and *105* encode 3 to 5 transcripts. Other genes only encode two cDNAs. In the case of *PtrZFP82*, out of the six cDNAs, Potri.005G164100.6 does not have a ZF motif because of alternative splicing. Out of the five cDNAs available for *PtrZFP54*, Potri.006G116500.2 and Potri.006G116500.5 do not encode a ZF motif. *PtrZFP105* also encodes five alternatively spliced cDNAs, but Potri.001G160700.3 and Potri.001G160700.5 do not have any ZF motifs. In the case of *PtrZFP86*, Potri.006G227600.1, Potri.006G227600.2 and Potri.006G227600.3 encode two, three and three fingers, respectively. Of the three transcripts representing *PtrZFP26*, Potri.012G143200.3 does not have any ZF motifs (S3 Table).

### Phylogenetic analysis, gene structure and conserved motifs of C2H2-ZF genes in *Populus*

**Fig 1:**
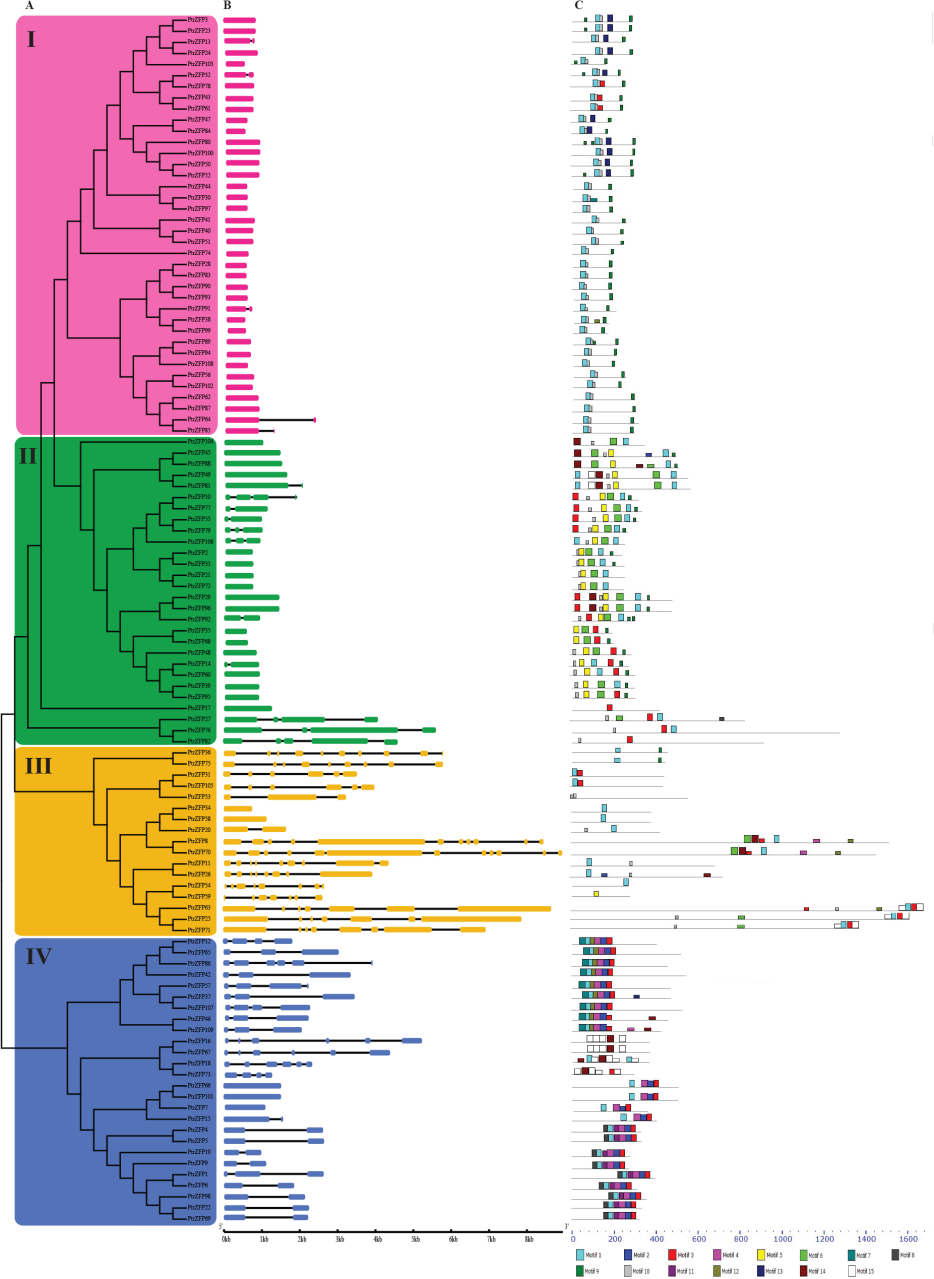
Phylogenetic relationships, gene structure and motif compositions of *Populus* C2HC2-ZF genes. **A.** Multiple alignment of 109 full-length amino acid sequences of *Populus* C2HC2-ZF genes, executed by ClustalX 1.83. The phylogenetic tree was constructed using MEGA 5.0 and the neighbor-joining method. Support values from a bootstrap analysis with 1,000 replicates are specified at each node. The four major phylogenetic subgroups, designated as I to IV, are marked with different colored backgrounds. **B.** Exon/intron structures of *Populus* C2HC2-ZF genes. Exon/intron structures were obtained from the Gene Structure Display Server. Exons and introns of each subgroup are represented by particular colored boxes and black lines, respectively. **C.** Schematic representation of the conserved motifs identified by MEME. Each colored box represents a motif and black lines represent non-conserved sequences.

To examine the phylogenetic relationships among the C2H2-ZF domain proteins in *Populus*, an unrooted phylogenetic tree was constructed from alignments of the full-length C2H2-ZFP sequences (Fig 1A). The 109 C2H2-ZF genes were classified into four groups (I, II, III and IV) containing 38, 28, 17, and 26 members, respectively. Almost 8000 pairs of paralogous genes are present in the *Populus* genome [55]. Based on the phylogenetic analysis, we identified 40 sister pairs, all of which had strong bootstrap support (>96%) (S4 Table).

Gene structural diversity and conserved motif divergence were a possible mechanism for the evolution of multigene families [44]. To gain further insights into the structural diversity of *Populus* C2H2-ZF genes, we analyzed the exon/intron organization in the full-length cDNAs with their corresponding genomic DNA sequences of individual C2H2-ZF genes in *Populus* (Fig 1B). Most closely related C2H2-ZF members within the same subgroups shared similar gene structures in terms of either intron numbers or exon lengths. For example, the C2H2-ZF group I and II genes had zero to three introns with exception of *PtrZFP82*. By contrast, the gene structure appeared to be more variable in subgroups III and IV, which had the largest number of exon/intron structure variants, with striking distinctions.

Putative protein motifs were predicted using MEME to further reveal the diversification of C2H2-ZF genes in *Populus* (Fig 1C). Fifteen distinct motifs were identified (S5 Table). Motifs 1, 3, 6, 14 and 15 represented different types of C2H2-ZF. These different types were found in all 109 *Populus* C2H2-ZFPs. Besides the C2H2-ZF domain, a known functional domain (Motif 9) was identified in the C2H2-ZFPs. Sixteen members contained the hexapeptide motif ‘DLELRL’ (Motif 9) in the C-terminal region. The hexapeptide motif acts as a repression domain and has been studied in *Arabidopsis* [56]. Furthermore, nine unidentified conserved motifs were observed. Only eight proteins (PtrZFP1, 4, 5, 6, 19, 22, 69 and 98) contained motif 11.

Different types of C2H2-ZFs have been identified in rice, *Arabidopsis* and petunia [10,13,14]. The first type includes a plant-specific conserved domain ‘QALGGH’, which is classified as the Q-type. The 62 identified Q-type C2H2-ZFPs in *Populus* were fewer than those present in the foxtail millet (97) [15], more than in bread wheat (47) [57] and similar to those in *Arabidopsis* (64) [13] and rice (65) [14]. Certain modifications of the Q-type ZF were designated as the M-type. The C-type does not include any conserved motif in the ZF region compared with the Q-type and M-type. Some of the ZFs contained highly conserved motifs in the C-type ZF and the flanking regions, and were named Z-type [14]. The details of the C2H2-ZF types are shown in S6 Table. *Populus* C2H2-ZFPs contained 1 to 6 ZF domains. Seventy-six proteins possess a single C2H2-ZF domain, which included three types of C2H2-ZF (Q-, Z- and C-types). Among them, 52 proteins contained a Q-type ZF, followed by 19 proteins with the C-type ZF and five proteins with the Z-type ZF. A significantly reduced number (23) contained two ZFs, among which 1 protein contained two C-type ZFs and five proteins contained two Q-type ZFs. For the others, two proteins contained a Q- and M-type ZF combination and 15 contained a C- and Z-type combination. There were six proteins were three-fingered proteins, of which two proteins contained all C-type ZFs. Only one protein had four ZFs, which was an M- and C-type combination. Two five-fingered proteins comprised C- and Z-type ZFs, but the number of C- and Z-type was different. The single 6-fingered protein contained all C-type ZFs (S7 Table).

### Chromosomal locations and duplications of *Populus* C2H2-ZF genes

**Fig 2:**
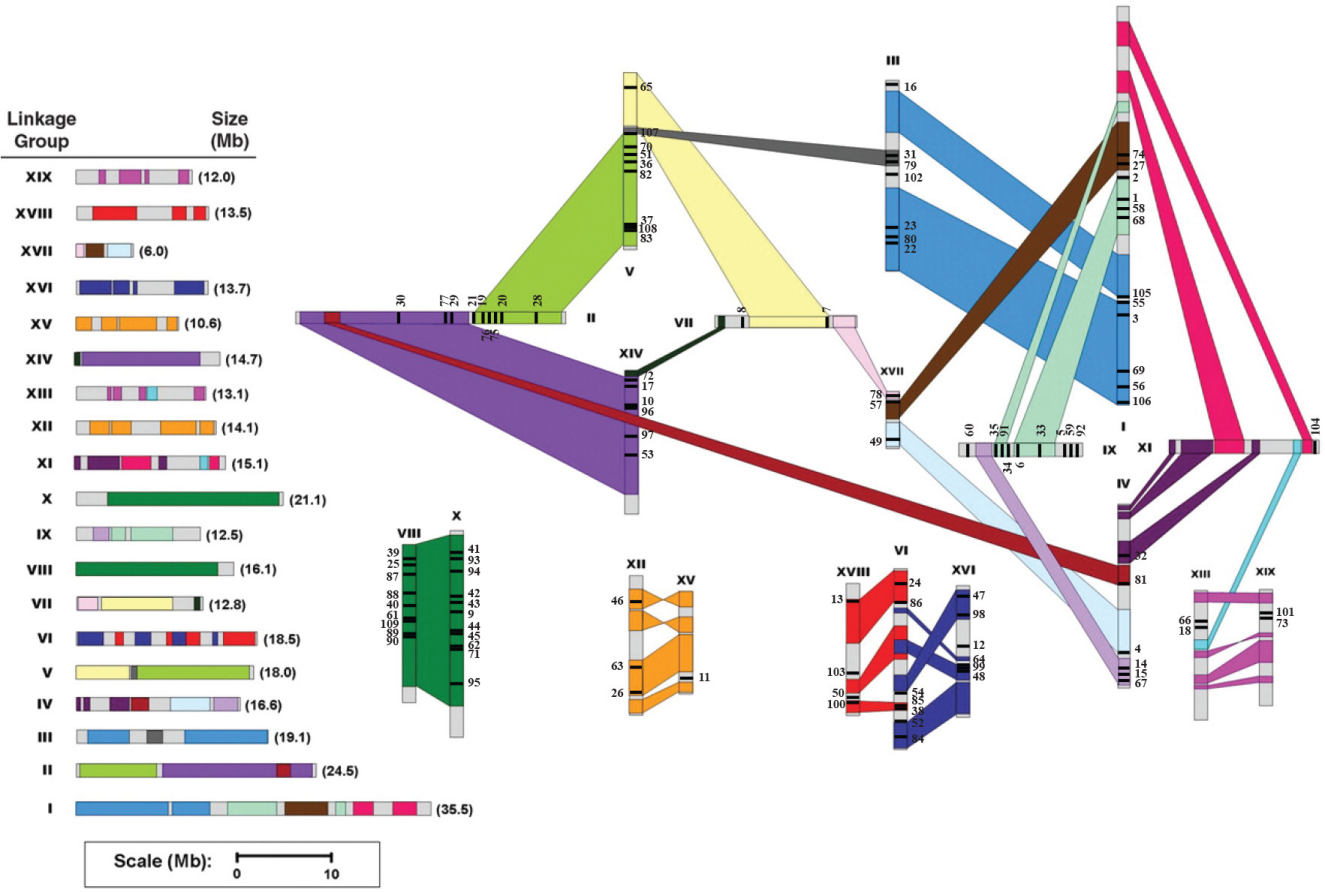
Chromosomal locations of *Populus* C2HC2-ZF genes. One hundred and nine C2HC2-ZF genes were mapped to 19 linkage groups (LG). A schematic view of chromosome reorganization caused by recent whole-genome duplication in *Populus* is shown (adapted from [32]). Segmental duplicated homologous blocks are indicated by the same color. The scale represents mega bases (Mb). The LG numbers are indicated above each bar.

*In silico* mapping of the gene loci showed that the 109 *Populus* C2H2-ZF genes were distributed across all 19 linkage groups (LGs). All of the *Populus* C2H2-ZF genes were distributed across the LGs. However, the distribution of *Populus* C2H2-ZF genes across the LGs was uneven. LG I has the largest number (12) of genes, followed by 11 on LG X. In contrast, only one or two genes were found on each of LG VII, LG XI, LG XIII, LG XV and LG XIX (Fig 2).

Previous studies revealed that the *Populus* genome has undergone at least three rounds of genome-wide duplication, followed by multiple segmental duplications, tandem duplications and transposition events, such as retrotransposition and replicative transposition [32]. In particular, the segmental duplication associated with the salicoid duplication event that occurred 65 million years ago contributed significantly to the expansion of many multigene families [54,58,59]. To determine the possible relationship between the C2H2-ZF genes and potential segmental duplications, we mapped the *Populus* C2H2-ZF genes to the duplicated blocks established in previous studies [32]. The distribution of C2H2-ZF genes relative to the corresponding duplicate blocks is shown in Fig 2. About 79% (86 of 109) of *Populus* C2H2-ZF genes were preferentially retained duplicates located in both duplicated regions. Eleven duplicated blocks only contained C2H2-ZF genes in one of the blocks and lacked duplicates in the corresponding block. By contrast, 23 C2H2-ZF genes were located outside any duplicated blocks, suggesting that dynamic changes may have occurred after segmental duplication, leading to the loss of some genes.

### Comparative analysis of the C2H2-ZF genes in *Populus* and rice

**Fig 3:**
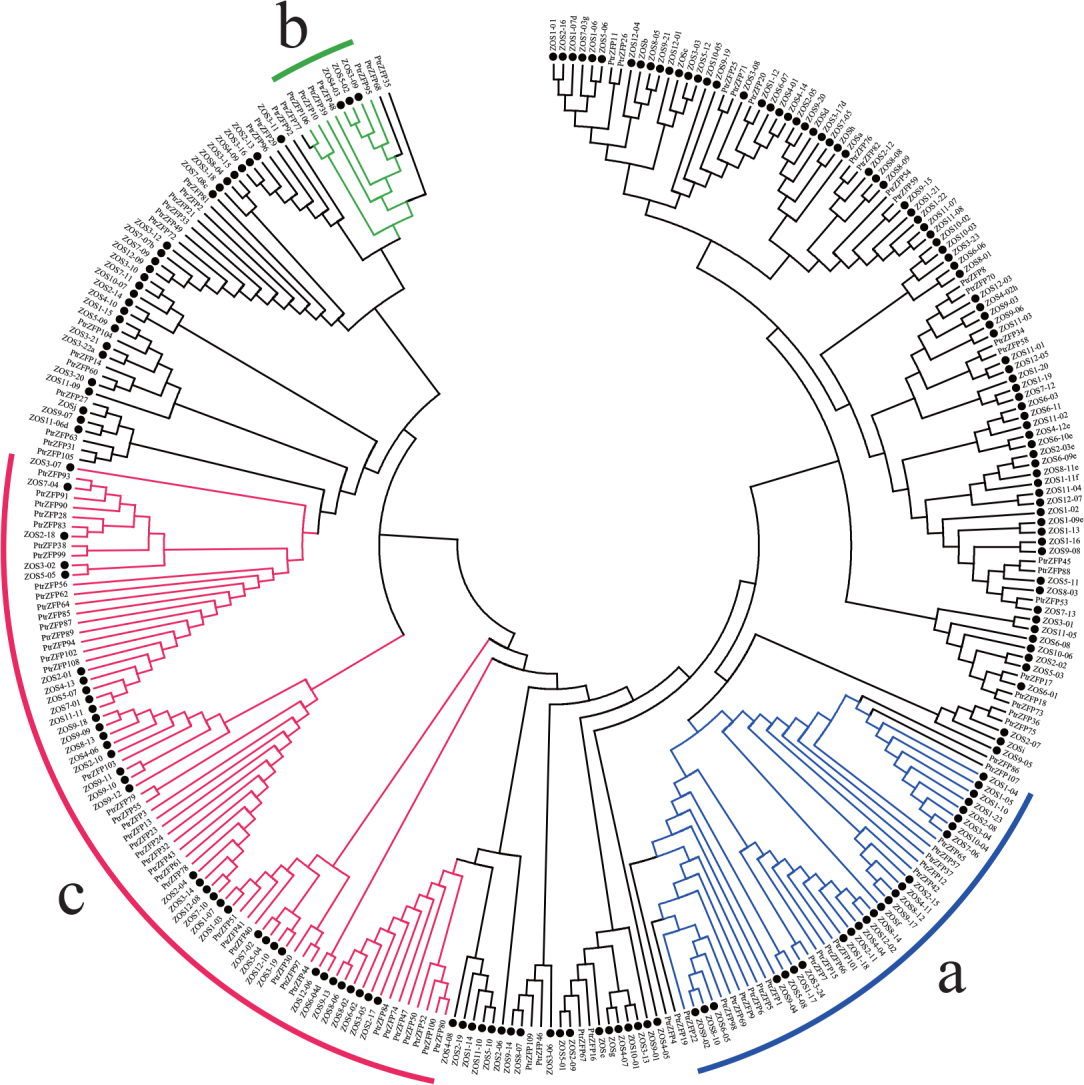
Phylogenetic tree of C2H2-ZF proteins from *Populus* and rice. One hundred and nine C2H2-ZF proteins in *Populus* and 189 in rice were aligned using Clustal X 1.83 and the phylogenetic tree was constructed using MEGA 5.0 by the neighbor-joining method. The Bootstrap value was 1,000 replicates. The rice proteins start with Z and are marked with a solid dot to distinguish them from the *Populus* C2HC2-ZF proteins. The three plant-specific clusters were designated as a, b and c, and indicated in a specific color.

To investigate the evolutionary relationship of the C2H2-ZF gene families, the full-length amino acid sequences of the 109 proteins from *P*. *trichocarpa* and 189 from rice (*Oryza sativa*) [14] were used to construct a phylogenetic tree. In rice, three plant-specific clusters including distinct types of C2H2-ZF have been identified compared with yeast and *Arabidopsis* [14]. In this study, we also observed the three clusters, named group a, b and c, comprising 16, 5 and 38 proteins, respectively (Fig 3). With few exceptions, group a proteins had a single C2H2-ZF domain, which contained Z2- to Z5-type ZFs; group b members possessed two consecutive Q-type ZFs, whereas the single Q-type ZF proteins were clustered in group c. The C2H2-ZFPs in groups a, b and c may be plant-specific. The other protein sequences, which mainly contained C- and Z1-type ZFs, appeared in plants and other species. According to the phylogenetic analysis of *Populus*, group a, b and c were also classified to group IV, II and I, respectively (Figs 1A and 3). It is noteworthy that some group a proteins from *Populus* not only had a Z2- to Z5-type ZF, but also contained a C-type ZF. Additionally, ZOS1-15/ZOS5-09/PtrZFP104, ZOS3-20/PtrZFP14/PtrZFP60 and ZOS9-07/ZOS11-06d/ZOSj/PtrZFP27 formed three small, unique clusters in the phylogenetic analysis. PtrZFP55 and 79, which contained two types of ZF (Q- and M1-type) were also clustered in group c (Fig 3).

### Promoter *cis*-element analysis

*Cis*-elements play key roles in the transcriptional regulation of genes that control abiotic stress responses, such as drought and heat stress [60]. Meanwhile, phytohormones, such as salicylic acid (SA), jasmonic acid (JA), ethylene (ET), and abscisic acid (ABA), are essential for plants’ ability to adapt to abiotic stresses, by inducing the interaction between TFs and corresponding *cis*-elements [61,62]. To identify putative *cis*-acting regulatory DNA elements in the C2H2-ZF genes, their promoter sequences (2 kb upstream of the translation start site) were obtained from the Phytozome v10.0 database, and the *cis*-elements of these 109 promoters were examined using the PlantCARE database. The C2H2-ZF gene family promoters harbored multiple *cis*-elements related to phytohormone and environmental stress signal responsiveness, such as MBS (MYB binding site, involved in drought-inducibility), HSE (heat stress-responsive element), ABRE (abscisic acid-responsive element), W-Box (WRKY binding site, involved in abiotic stress responsiveness), ERE (ethylene-responsive element) and TCA-element (salicylic acid-responsive element) (S8 Table). The majority of C2H2-ZF genes contained *cis*-elements related to phytohormone and environmental stress signal responsiveness. *PtrZFP4* and *92* have nine *cis*-elements (ABRE, CGTCA-motif, ERE, G-Box, HSE, MBS, TCA-element, TGACG-motif and W-Box), whereas *PtrZFP17*, *40* and *94* have only two *cis*-elements (S9 Table).

### Gene Ontology (GO) Annotation

**Fig 4:**
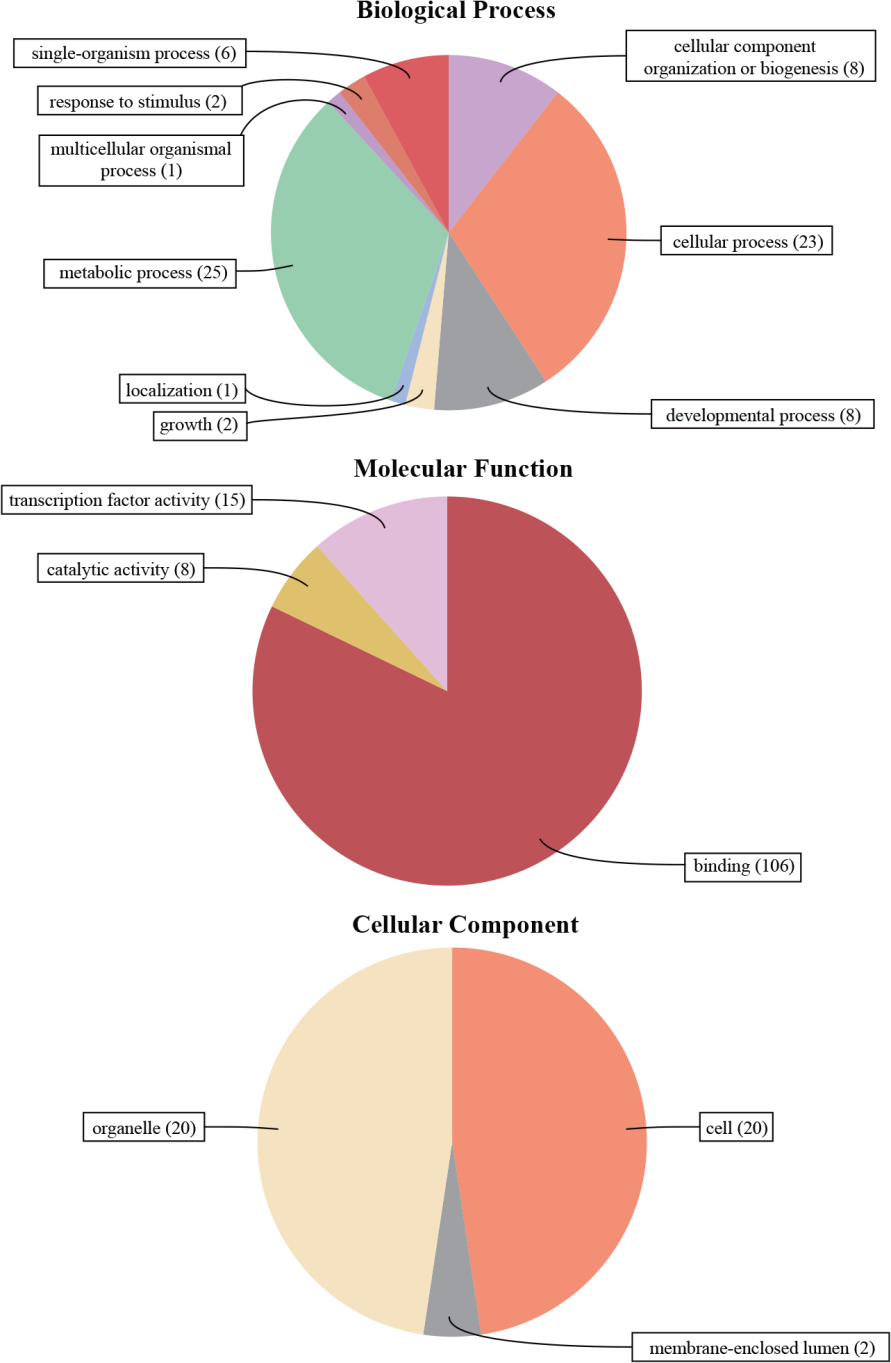
Gene Ontology (GO) results for *Populus* C2HC2-ZF proteins. GO analysis of 109 C2HC2-ZFP sequences predicted for their involvement in biological processes, molecular functions and cellular components. For the results presented as detailed bar diagrams, see S10 Table.

The biological processes, molecular functions and cellular components of *Populus* C2H2-ZF genes were investigated based on the putative assignment of Gene Ontology (GO) terms using Blast2GO v3.0 (Fig 4, S10 Table). The results showed that the 109 C2H2-ZF genes putatively participated in diverse biological processes. Of the nine terms of biological processes defined by Blast2Go, most *Populus* C2H2-ZFPs were predicted to function in the metabolic process (~29%) and the cellular process (~27%), followed by cellular component organization or biogenesis (~9%) and the developmental process (~9%). Molecular function prediction indicated that all 109 C2H2-ZFPs were annotated as small molecule or/and ion binding (~82%), which is in accordance with the molecular role of C2H2-ZFP in DNA and metal ion binding. In addition, some C2H2-ZFPs were involved in transcription factor activity (~12%) and catalytic activity (~6%). In addition, cellular component prediction showed that 20 *Populus* C2H2-ZFPs were localized in the cell part (~48%) and organelle (~48%), respectively. Only two C2H2-ZFPs were membrane-enclosed lumen-localized (~4%) (Fig 4, S10 Table).

### Expression profile of *Populus* C2H2-ZF genes in different tissues or organs

**Fig 5:**
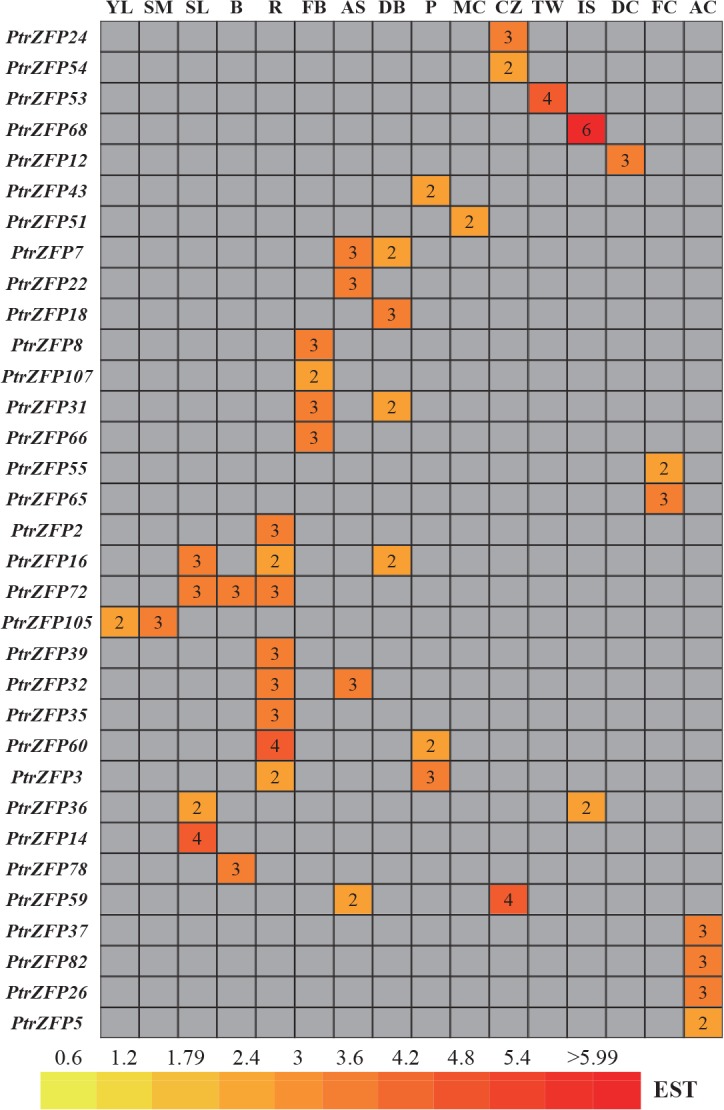
*In silico* EST analysis of *Populus* C2HC2-ZF genes. EST frequency for each gene was calculated by evaluating its EST representation among 16 cDNA libraries accessible using the exNorthern tool at the PopGenIE v2 database. The number in the box represents the frequencies of EST counts in different libraries. YL: young leaves, SM: shoot meristem, SL: senescing leaves, B: bark, R: roots, FB: flower buds, AS: apical shoot, DB: dormant buds, P: petioles, MC: male catkins, CZ: cambial zone, TW: tension wood, IS: imbibed seeds, DC: dormant cambium, FC: female catkins, AC: active cambium.

Publicly available expressed sequence tags (ESTs) are considered useful tools to study gene expression profiles using the Digital Northern tool [63]. We first carried out a preliminary analysis of C2H2-ZF gene expression across different tissues and organs by counting the frequencies of ESTs in different *Populus* cDNA libraries. A complete search of the digital expression profiles in PopGenIE yielded 33 *Populus* C2H2-ZF genes in the cDNA libraries. As illustrated in Fig 5, the C2H2-ZF gene family had a varied expression pattern across various developmental stages of tissues and organ. Among the 33 genes, one showed the highest transcript accumulation in young leaves, one in the shoot meristem, four in senescing leaves, two in bark, eight in roots, four in flower buds, four in apical shoots, four in dormant buds, three in petioles, one in male catkins, three in the cambial zone, one in tension wood, two in imbibed seeds, one in the dormant cambium, two in female catkins and four in the active cambium. A putative paralogous gene (*PtrZFP54/59*) had the highest transcript accumulation in the cambial zone. However, the expression patterns of a few gene pairs, including *PtrZFP14/60*, *PtrZFP31/105*, *PtrZFP35/68*, *and PtrZFP37/107*, were significantly different, although they were paralogous genes (Figs 1 and 5). These results were similar to a previous study of the SPL gene family in *Populus* [64]. Moreover, *PtrZFP53* was highly expressed in tension wood. However, some genes (*PtrZFP8*, *31*, *51*, *55*, *65*, *66*, and *107*) were mainly expressed during flower development.

### Expression profiles of *Populus* C2H2-ZF genes under various stresses

**Fig 6:**
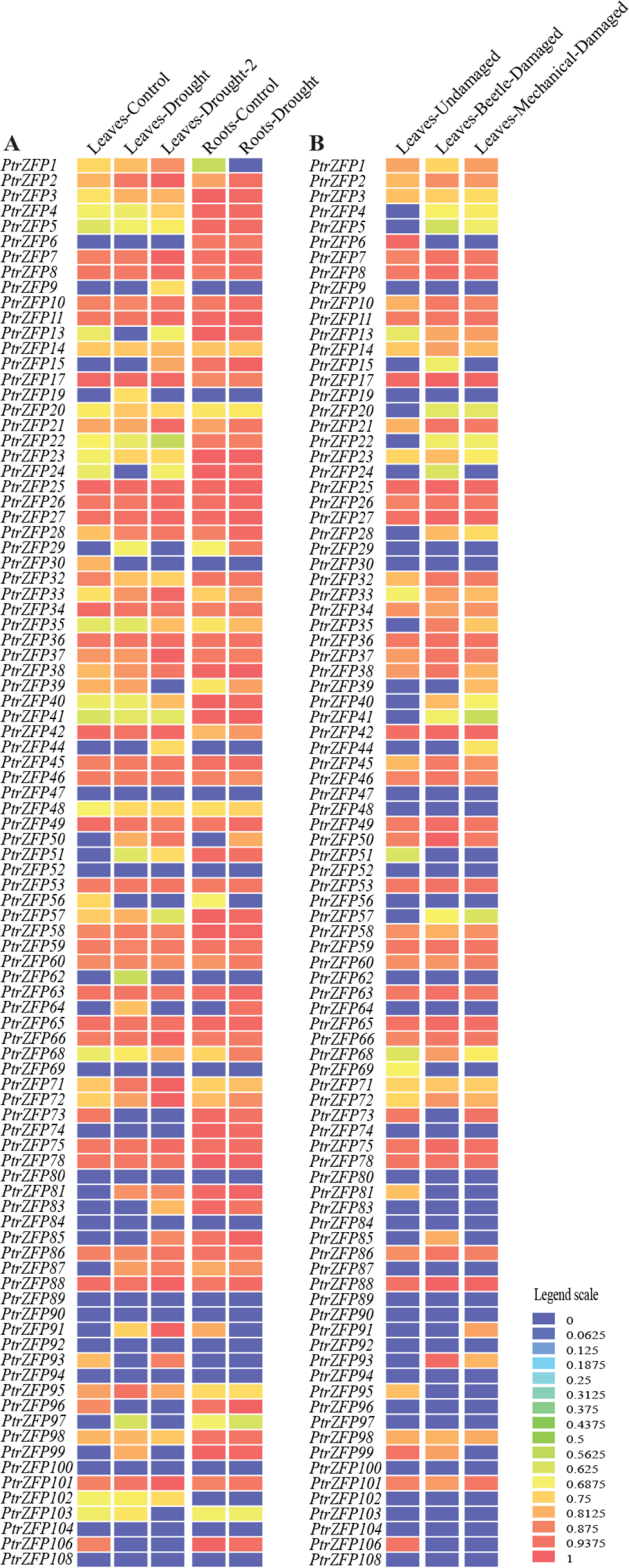
Differential expression of *Populus* C2HC2-ZF genes under different stress conditions. The heatmap was visualized using the exHeatmap tool in the PopGenIE v2 database. Blue and red indicate low and high levels of transcript abundances, respectively. **A.** Heatmap showing 92 *Populus* C2HC2-ZF genes in leaves and roots under drought stress. **B.** Heatmap showing 92 *Populus* C2HC2-ZF genes under beetle and mechanical stress.

Drought is the main environmental stress encountered by most land plants during their life span. To gain further insights into the potential roles of *Populus* C2H2-ZF genes in drought tolerance, we analyzed the expression profiles of *Populus* C2H2-ZF genes in response to drought stress using the publicly available heatmap data. The heatmap data can be directly downloaded using the accession numbers of genes via the exHeatmap tool in the PopGenIE v2 database (http://www.popgenie.org/). According to the present data set, 44 genes were upregulated in leaves and 20 genes were upregulated in roots under drought stress (Fig 6A). Thirteen genes were upregulated in leaves and roots. Thus, the site of activity of the gene was also different under drought stress. This result was consistent with previous analyses, which revealed that the C2H2-ZF gene family contributed to variation in the drought response [26,28]. In other biotic and abiotic stresses, beetle damage induced the upregulation of 29 C2H2-ZF genes and mechanical wounding induced the upregulation of 22 genes (Fig 6B). Notably, *PtrZFP2*, *21*, *28*, *33*, *35*, *68*, and *72* were upregulated by drought, beetle and mechanical damage (Fig 6A and 6B).

### Validation of C2H2-ZF gene expression under drought, heat and salt stress by qRT-PCR

**Fig 7:**
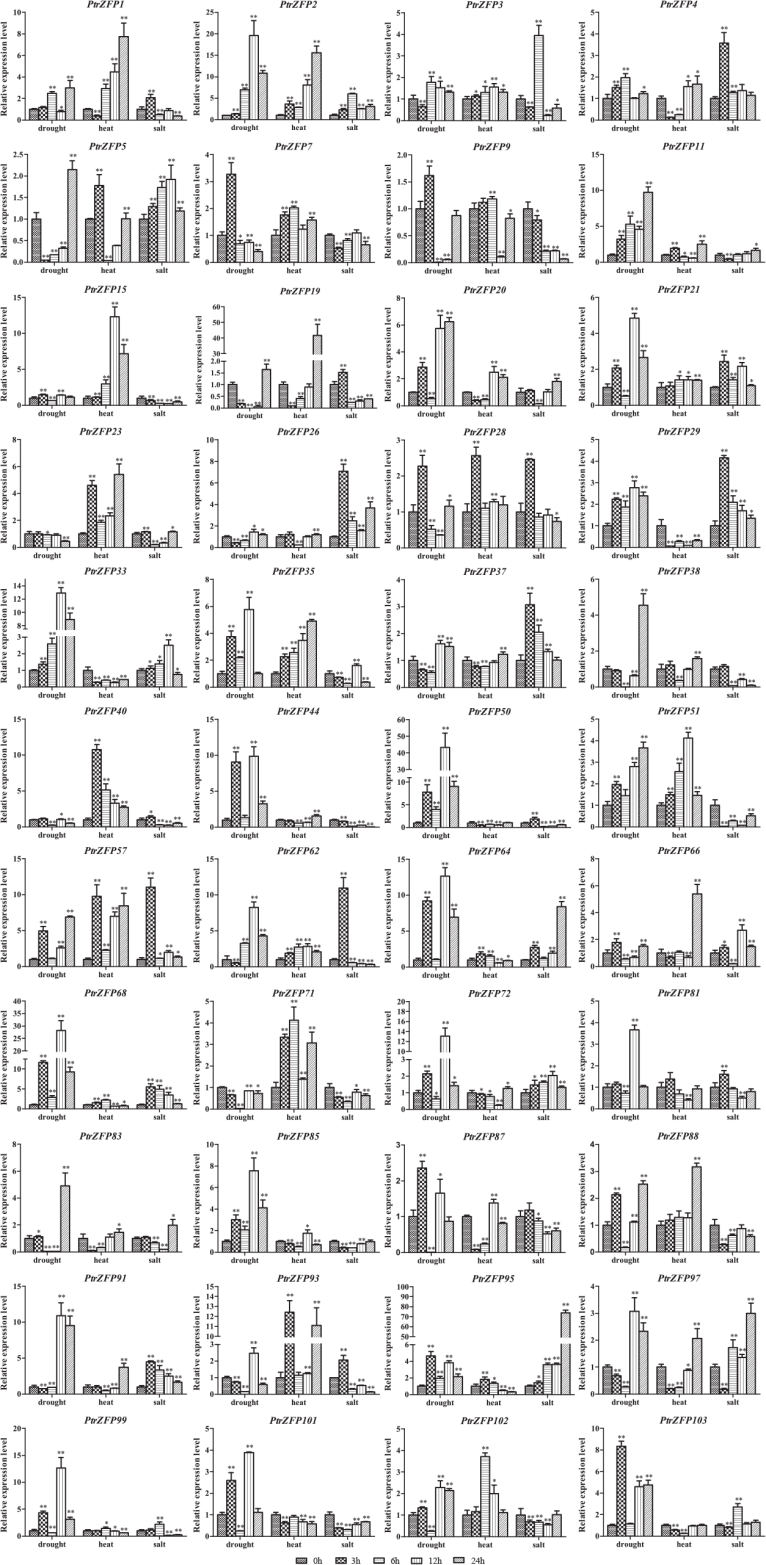
Expression analysis of 44 selected C2HC2-ZF genes in leaves under drought, heat and salt stresses using qRT-PCR. The relative mRNA abundance of 44 selected C2HC2-ZF genes was normalized with respect to the reference gene (*Actin1*). The *x*-axis represents time points after stress treatments. Error bars represent the standard deviations from three biological replicates. Asterisks indicate stress treatment groups that showed a significant difference in transcript abundance compared with the control group (* *P* < 0.05, ** *P* < 0.01).

**Fig 8:**
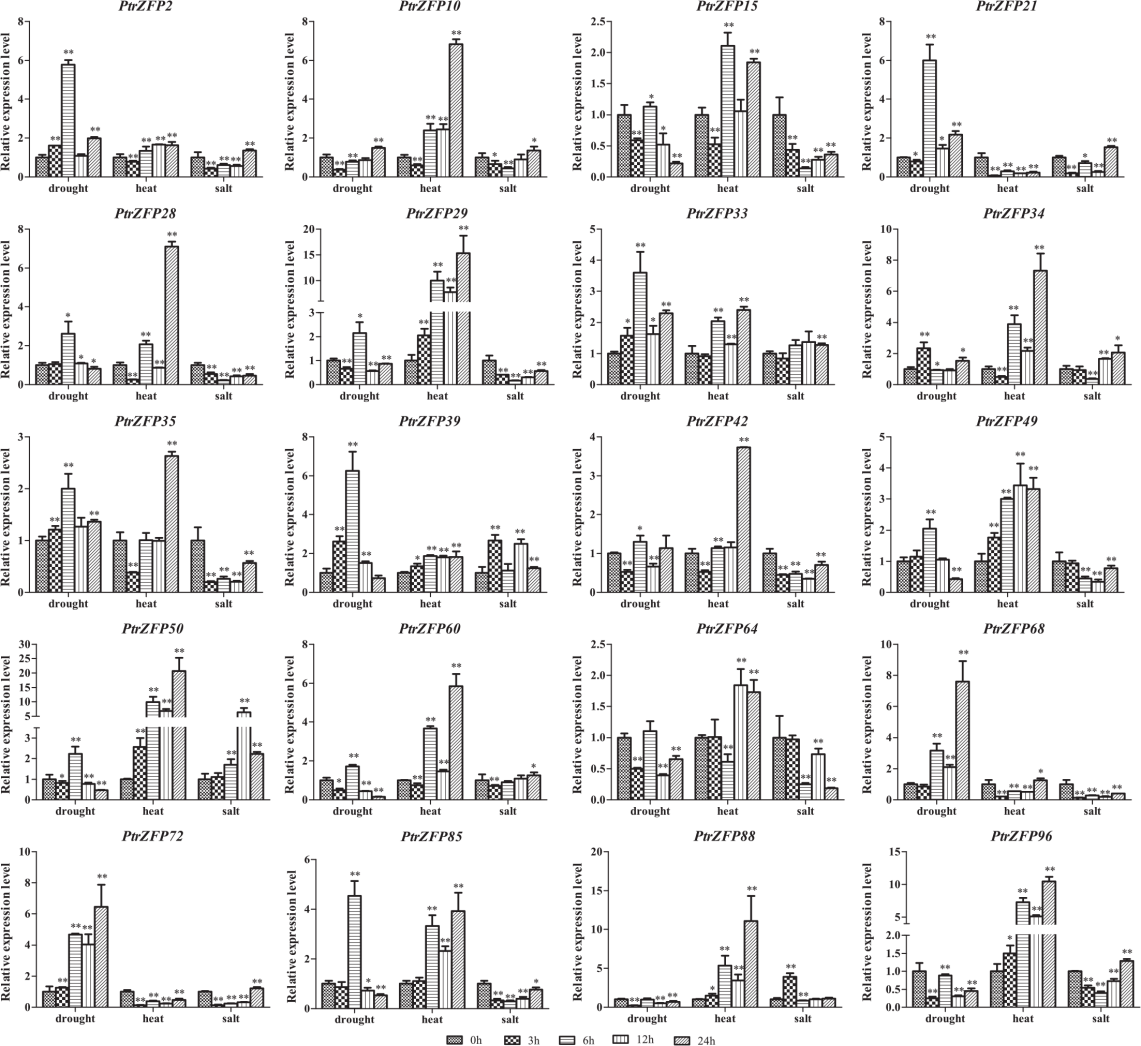
Expression analysis of 20 selected C2HC2-ZF genes in roots under drought, heat and salt stresses using qRT-PCR. The relative mRNA abundance of 20 selected C2HC2-ZF genes was normalized with respect to the reference gene (*Actin1*). The *x*-axis represents time points after stress treatments. Error bars represent the standard deviations from three biological replicates. Asterisks indicate stress treatment groups that showed a significant difference in transcript abundance compared with the control group (* *P* < 0.05, ** *P* < 0.01).

To verify the expression profiles of *Populus* C2H2-ZF genes obtained by the heatmap analysis, qRT-PCR was performed for 51 selected genes (44 genes upregulated in leaves and 20 genes upregulated in roots, including 13 genes upregulated both in leaves and roots) under drought stress. The genes up- or downregulated by more than 1.5-fold were considered significantly differentially expressed. The results were broadly consistent with the heatmap data: 41 genes were induced, one gene (*PtrZFP71*) was suppressed and two genes (*PtrZFP23* and *40*) showed no change in leaves under drought stress (Fig 7). In roots, 16 genes were induced, one gene (*PtrZFP88*) was suppressed and three gene (*PtrZFP64*, *95* and *96*) were not influenced by drought (Fig 8). Notably, *PtrZFP2*, *11*, *29*, *33*, *50*, *68*, *85* and *95* were upregulated significantly at all time points in leaves, whereas *PtrZFP33* and *72* were upregulated significantly at all time points in roots (Figs 7 and 8). In addition, *PtrZFP2*, *15*, *21*, *28*, *29*, *33*, *35*, *50*, *68* and *72* not only exhibited a high expression level in leaves, but were also upregulated in roots. The results were consistent with those observed in heatmap analysis (Figs 6A, 7 and 8); however, the levels of upregulation were slightly different.

Subsequently, the expression profiles of the 51 selected genes were further analyzed under heat and salt stress. Under heat stress treatment, 34 genes were induced, two genes (*PtrZFP29* and *33*) were suppressed and eight genes (*PtrZFP9*, *26*, *37*, *50*, *72*, *81*, *101* and *103*) were unchanged in leaves (Fig 7). Seventeen genes were induced, two genes (*PtrZFP21* and *72*) were suppressed and one gene (*PtrZFP68*) was not influenced in roots after heat treatment (Fig 8). In leaves, 10 genes (*PtrZFP2*, *3*, *15*, *23*, *35*, *40*, *51*, *57*, *62* and *71*) were upregulated significantly at all time points, whereas six genes (*PtrZFP29*, *39*, *49*, *50*, *88* and *96*) were upregulated significantly at all time points in roots. *PtrZFP2*, *15*, *28*, *35*, *64*, *85* and *88* showed high expression levels in leaves and roots. *PtrZFP19* was strongly upregulated (>40-fold) at 24 h after heat treatment (Figs 7 and 8). Under salt stress, 30 genes were induced, seven genes (*PtrZFP9*, *15*, *44*, *51*, *71*, *88* and *101*) were suppressed and seven genes (*PtrZFP7*, *23*, *38*, *40*, *85*, *87* and *102*) were not affected in leaves (Fig 7). Five genes were induced, seven genes (*PtrZFP15*, *28*, *29*, *35*, *42*, *68* and *85*) were suppressed and eight genes (*PtrZFP10*, *33*, *49*, *60*, *64*, *72*, *85* and *96*) were unchanged in roots after NaCl treatment (Fig 8). Among these genes, *PtrZFP2*, *5*, *21*, *26*, *29*, *57*, *68*, *72* and *91* were upregulated significantly at all time points in leaves; no genes were upregulated significantly at all time points in roots. Notably, *PtrZFP2*, *21* and *50* displayed significantly high expression levels in leaves and roots. *PtrZFP95* was greatly upregulated (>70-fold) at 24 h after NaCl treatment (Figs 7 and 8).

In summary, our results showed that these candidate C2H2-ZF genes responded positively to more than one kind of stress. For example, among the 41 drought-inducible genes in leaves, 32 were upregulated by heat and 28 by salt. Meanwhile, among the 16 drought-inducible genes in roots, 15 were upregulated by heat and four by salt. *PtrZFP2* demonstrated a significantly positive response to all three stresses at some time points after treatment, whether in leaves or roots. It is also noteworthy that all upregulated gene promoter sequences contained *cis*-elements related to phytohormone and abiotic stresses, such as ABRE, G-box, HSE, MBS, TCA-element and W-Box. However, we also observed that some genes were downregulated under various stresses: *PtrZFP71* was suppressed in leaves by drought and salt, whereas *PtrZFP29* was suppressed in leaves by heat and suppressed in roots by salt.

## Discussion

Preliminary analysis of the C2H2-ZF gene family has been performed in the model plants *Arabidopsis* and rice [13,14]. However, this family has not previously been studied in *P*. *trichocarpa*, a model forest tree. In this report, we identified 109 full-length C2H2-ZF genes in the *P*. *trichocarpa* genome, each of which contains at least one C2H2-ZF motif. The lengths of these sequences varied significantly, implying a high degree of complexity among the C2H2-ZF genes. About 94% (103 of 109) of the C2H2-ZF genes were predicted as nuclear proteins, whereas the other six genes were predicted to be located in the chloroplast or cytoplasm. This suggested that these genes had special functions compared with the other members in this family. Furthermore, based on the WoLF PSORT analyses, we were able to roughly determine their gene localizations; however, experimental verification is required for more accurate localization. Based on the phylogenetic analysis, we identified 40 paralogous pairs among the 109 *Populus* C2H2-ZF genes. Among the 40 gene pairs, 39 belong to the same group and identical subcellular localizations were predicted for their proteins (Fig 1A, S2 and S4 Tables). However, PtrZFP35 and PtrZFP68 were predicted to be localized in the chloroplast and nucleus, respectively, even though they were both placed in group II. These results indicated that the same phylogenetic grouping based on sequence similarity did not necessarily correspond to the same subcellular localization. Therefore, homologous genes may show differences in gene function and signal transduction. These results were similar to those of a previous analysis of the C3HC4-type RING finger gene family in *Populus* [65].

Exon–intron increase or decrease can be caused by integrations and realignments of gene fragments. Therefore, gene structural variation plays a major role in the evolution of gene families [66]. The current study provided an example of such diversification in the form of a C2H2-ZF gene (*PtrZFP34*) with only one exon, whereas other genes in the same phylogenetic group (group III) have seven or eight exons. Moreover, *PtrZFP70* has 12 exons, whereas *PtrZFP20*, which is similar to *PtrZFP70*, only has two exons. Previous studies showed that many plant-specific Q-type C2H2-ZFPs play important roles in diverse environmental stress responses, as well as in various plant developmental and physiological processes [14,57]. In this study, about 57% (62 of 109) of C2H2-ZFPs from *Populus* had Q-type ZF domains, which was a larger number compared with other experimental models such as *Arabidopsis* (36%) and rice (34%). This result suggested that Q-type C2H2-ZFPs are more important for woody plants. However, only eight proteins (PtrZFP1, 4, 5, 6, 19, 22, 69 and 98) contained motif 11, which suggested that these proteins may have special functions. The similar gene structures and conserved motifs of C2H2-ZF genes and proteins in the same subfamilies may provide additional support for the phylogenetic analysis. Conversely, the differences in gene structure and motif composition among different subfamilies indicated that they might be functionally divergent.

Gene duplication events, including tandem and segmental duplication, play an important role in genomic expansions and realignments [67,68]. Gene duplication has been reported for many plant transcription factor gene families, such as the NAC, CCCH and HD-ZIP families [44,69,70]. To verify this, the mechanisms involved in the expansion of C2H2-ZF members in *Populus* were examined. Among the 40 sister pairs, only one pair (*PtrZFP37/107*) of the C2H2-ZF genes appeared to have undergone tandem duplication, based on their more than 96% similarity at the amino acid level. By contrast, 39 segmental duplication events were identified, suggesting the existence of low-tandem and high-segmental repetitions in the C2H2-ZF gene family. The result was similar to that observed for the WRKY duplications in *P*. *trichocarpa* [71].

In this study, we compared the members of the C2H2-ZF gene family in *Populus* with those in rice and found that three groups were plant-specific clusters including distinct types of C2H2-ZF. Compared with the rice, some proteins in the *Populus* group a had added a C-type ZF domain, which suggested that woody plants might have undergone a series of changes during the evolutionary process. In addition, three small unique clusters (ZOS1-15/ZOS5-09/PtrZFP104, ZOS3-20/PtrZFP14/PtrZFP60 and ZOS9-07/ZOS11-06d/ZOSj/PtrZFP27) may have undergone independent evolutionary trajectories from the other clusters. Two 2-fingered proteins (PtrZFP55 and 79) were assigned to group c, which suggested that they might share a common origin with other single Q-type finger members.

It is accepted that c*is*-elements play key roles in the transcriptional regulation of genes controlling various abiotic stress and phytohormone responses. Plant hormones play central roles in the ability of plants to adapt to changing environments. Many abiotic stress-related and phytohormone-related *cis*-elements, including MBS, HSE, ABRE, W-Box, ERE and TCA-elements, have been identified [72–74]. All of these *cis*-elements were observed in the present study (S4 Table). Each gene of this family contained at least two *cis*-elements related to phytohormone or abiotic stress signal responsiveness. *PtrZFP17*, *40* and *94* have only two *cis*-elements, which suggested that this gene might not be associated with abiotic stress. By contrast, *PtrZFP4* and *92* have nine *cis*-elements, which suggested that these genes might have important functions under different abiotic stresses. These results were consistent with expression profiles of *Populus* C2H2-ZF genes under various stresses. *PtrZFP17* and *94* showed no change under drought and mechanical damage, whereas *PtrZFP4* was upregulated in leaves at some time points after drought, heat and salt treatment.

Evidence suggested that C2H2-ZF transcription factors are involved widely in the integration and development of many organs and tissues, such as seed maturation [75], floral development [76], secondary metabolism and cell wall structure [77]. Using *in silico* analysis, *PtrZFP105*, identified in the present study, was observed to be preferentially expressed in young leaves, suggesting a role in the regulation of adaxial leaf fate. *PtrZFP2*, *3*, *16*, *32*, *35*, *39*, *60* and *72* showed high expression levels in roots, indicating that these genes may affect root development. The putative paralogous genes (*PtrZFP54/59*) showed high transcript accumulation in the cambial zone, suggesting that they are involved in the same regulation network of biological processes. *PtrZFP8*, *31*, *51*, *55*, *65*, *66* and *107* were mainly expressed in flower buds, male catkins and female catkins, indicating that these genes might be closely related to flower differentiation and floral organ formation. Notably, *RID1* (LOC_Os10g28330), a *PtrZFP65* orthologous gene, acts as a master switch from vegetative to floral development in rice [76]. This suggested that *PtrZFP65* might have functions similar to *RID1* during floral development in *P*. *trichocarpa*. Interestingly, *PtrZFP107* was preferentially expressed in *Populus* flower buds, whereas *ENY* (AT5G66730), an *Arabidopsis* ortholog of *PtrZFP107*, is involved in seed maturation [75]. This result suggested that their functions might vary in different plant species. In addition, *PtrZFP53* was abundantly expressed in tension wood. *Populus*, as an important tree species for a large variety of wood-based products, produces abundant wood (secondary cell wall) compared with herbaceous plants. Tension wood is mainly characterized by abnormal fibers that are poorly lignified and have an additional thick layer in the secondary cell wall [78]. Therefore, tension wood is involved in the formation of wood. Considering the high expression level of *PtrZFP53* in tension wood, we hypothesized that the C2H2-ZF gene family might contribute to wood formation. This conclusion is consistent with previous research on cotton fiber quality [77]. *Populus* C2H2-ZF genes may also be involved in other biological processes, such as shoot and cambium development, hinted at by their abundant expression in the shoot meristem and active cambium. Gene ontology annotation also supported this hypothesis. Determination of the functions and mechanisms of action of the identified C2H2-ZF members requires further experiments.

In the life cycle of a tree, growth and productivity are frequently threatened by environmental stresses, such as beetle damage, drought, heat and high salinity. These stresses may cause fatal damage to trees [79–81]. However, many stress-related genes are induced to help plants adapt to these biotic and abiotic stresses. In this study, *PtrZFP2*, *21*, *28*, *33*, *35*, *68* and *72* were upregulated in drought, beetle and mechanical damage, suggesting that these genes play essential roles under multiple stresses. In plants, leaves and roots are the most important organs for resisting abiotic stress. Plant leaves provide an adaptive mechanism for plants undergoing abiotic stress by increasing stomatal closure, decreasing transpiration rate and reducing the leaf area [82,83]. Roots can sense soil changes in the surrounding environment under abiotic stress conditions, and send a series of signals to the leaves and shoots to reduce root damage and maintain plant growth in spite of water shortage [84]. Based on *in silico* analysis and our qRT-PCR analysis, 32 genes were upregulated in leaves in the comparison between drought and heat, 28 genes were upregulated in leaves in the comparison between drought and salt, 15 genes were upregulated in roots in the comparison between drought and heat, and four genes were upregulated in roots in the comparison between drought and salt. *PtrZFP2* acts as a positive regulator under all three stresses in leaves and roots. Notably, *ZFP252* (GenBank: AAO46041.1), a *PtrZFP2* orthologous gene, can enhance drought and salt tolerance in rice [85]. Similar results were found in potato [86]. Our study found that *PtrZFP2* was also involved in response to heat stress. These results indicated that C2H2-ZF genes might be involved in the substantial common regulatory systems or cross-talk triggered by different stresses. Many abiotic stresses ultimately result in dehydration and osmotic imbalance of plant cells; therefore, it is not surprising that there is a large overlap of genes induced by drought, heat and salt stresses [81,87]. These genes may have shared roles in two or more stresses. Interestingly, some drought-upregulated genes (*PtrZFP29* and *33*) were downregulated by heat stress, which indicated that two sets of C2H2-ZF genes are involved in response to drought and heat stress, respectively. The huge expression differences suggest that these genes carry out different physiological and biochemical functions to adapt to complicated challenges. Therefore, it would be interesting to undertake further functional studies of these C2H2-ZF genes to establish the interactions among the particular pathways that are activated during the drought, heat and salt stress responses.

## Conclusions

In the present study, we performed a comprehensive analysis of phylogenetic relationships, chromosomal locations, gene structures, conserved motifs, *cis*-elements and expression profiles of the C2H2-ZF gene family in *Populus*. One hundred and nine full-length C2H2-ZF genes in the *Populus* genome were identified, which were clustered phylogenetically into four distinct subfamilies. The genes were non-randomly distributed across 19 LGs, and segmental duplications had contributed significantly to the expansion of the *Populus* C2H2-ZF gene family. *Cis*-elements in the C2H2-ZF genes provided clues to their functions and expression in specific tissues or organs, as well as under different biotic and abiotic stresses. We identified a subset of *Populus* C2H2-ZF genes with putative functional roles in drought, heat and salt responses. The information obtained in the current study could help to select appropriate candidate genes for further functional characterization to unravel their divergent roles.

## Supporting Information

S1 Table Primers for qRT-PCR of 51 selected C2HC2-ZF genes were designed using Primer Premier 5 (F represents a forward primer, R represents a reverse primer). (XLSX)

S2 Table List of 109 C2HC2-ZF genes identified in *P*. *trichocarpa* and their sequence characteristics (aa, amino acids; Da, Dalton). (XLSX)

S3 Table Alternative splicing of *Populus* C2HC2-ZF genes. (XLSX)

S4 Table Divergence between C2HC2-ZF genes pairs in *Populus*. Gene pairs were identified at the terminal nodes (> 96% identical) of the phylogenetic tree shown in Fig 1A. Synonymous (Ks) and nonsynonymous substitution (Ka) rates are presented for each pair. Gene pairs created by tandem duplication or segmental duplication are indicated in the table.
(XLSX)

S5 Table Motif sequences of C2HC2-ZF genes identified in *P*. *trichocarpa*. (XLSX)

S6 Table Types of C2H2-ZF motifs. (XLSX)

S7 Table Numbers and types of *Populus* C2H2-ZFPs. (XLSX)

S8 Table Phytohormone and abiotic stress related *cis*-elements. (XLSX)

S9 Table Abiotic stress and phytohormone response elements in *Populus* C2H2-ZFP promoters. (XLSX)

S10 Table Details of the Gene Ontology annotation of *Populus* C2HC2-ZFP sequences. (XLSX)

S1 Text Information for 109 C2HC2-ZF genes. A complete list of the 109 C2HC2-ZF gene sequences of *P*. *trichocarpa* identified in this study. Genomic DNA and amino acid sequences were downloaded from Phytozome v10.0 (http://phytozome.jgi.doe.gov/pz/portal.html) and NCBI (http://www.ncbi.nlm.nih.gov/) databases.
(TXT)
